# Identification of a necroptosis-related prognostic gene signature associated with tumor immune microenvironment in cervical carcinoma and experimental verification

**DOI:** 10.1186/s12957-022-02802-z

**Published:** 2022-10-17

**Authors:** Kai Sun, Cheng Huang, Jing-zhang Li, Zhan-xiong Luo

**Affiliations:** Department of Oncology, Liuzhou People’s Hospital, Guangxi Zhuang Autonomous Region, Liuzhou, 545001 China

**Keywords:** Cervical carcinoma, Necroptosis-related genes, Prognostic signature, Tumor microenvironment infiltration

## Abstract

Cervical carcinoma (CC) has been associated with high morbidity, poor prognosis, and high intratumor heterogeneity. Necroptosis is the significant cellular signal pathway in tumors which may overcome tumor cells’ apoptosis resistance. To investigate the relationship between CC and necroptosis, we established a prognostic model based on necroptosis-related genes for predicting the overall survival (OS) of CC patients. The gene expression data and clinical information of cervical squamous cell carcinoma and endocervical adenocarcinoma (CESC) patients were obtained from The Cancer Genome Atlas (TCGA). We identified 43 differentially expressed necroptosis-related genes (NRGs) in CESC by examining differential gene expression between CESC tumors and normal tissues, and 159 NRGs from the Kyoto Encyclopedia of Genes and Genomes (KEGG) database. Gene ontology (GO) and KEGG enrichment analysis illustrated that the genes identified were mainly related to cell necrosis, extrinsic apoptosis, Influenza A, I − kappaB kinase/NF − kappaB, NOD − like receptor, and other signaling pathways. Subsequently, least absolute shrinkage and selection operator (LASSO) regression and univariate and multivariate Cox regression analyses were used to screen for NRGs that were correlated with patient prognosis. A prognostic signature that includes CAMK2A, CYBB, IL1A, IL1B, SLC25A5, and TICAM2 was established. Based on the prognostic model, patients were stratified into either the high-risk or low-risk subgroups with distinct survival. Receiver operating characteristic (ROC) curve analysis was used to identify the predictive accuracy of the model. In relation to different clinical variables, stratification analyses were performed to demonstrate the associations between the expression levels of the six identified NRGs and the clinical variables in CESC. Immunohistochemical (IHC) validation experiments explored abnormal expressions of these six NRGs in CESC. We also explored the relationship between risk score of this necroptosis signature and expression levels of some driver genes in TCGA CESC database and Gene Expression Omnibus (GEO) datasets. Significant relationships between the six prognostic NRGs and immune-cell infiltration, chemokines, tumor mutation burden (TMB), microsatellite instability (MSI), and immune checkpoints in CESC were discovered. In conclusion, we successfully constructed and validated a novel NRG signature for predicting the prognosis of CC patients and might also play a crucial role in the progression and immune microenvironment in CC.

**Supplementary Information:**

The online version contains supplementary material available at 10.1186/s12957-022-02802-z.

## Introduction

Cervical carcinoma (CC) is a major disease in women, with 604,000 new cases worldwide in 2020 [1,2]. In 2021, it was estimated that approximately 13,800 people were diagnosed with CC along with 4290 CC-related deaths in the USA [3]. CC is an illness characterized by high morbidity and mortality rate [4]. Although advancements in surgical methods, chemotherapy, radiotherapy, and targeted therapy for CC have already been made, a large proportion of CC patients still did not benefit from currently available treatment strategies [5]. Since CC has shown a high tumor mutation burden, immunotherapy has become a crucial therapeutic strategy during the progressive stage of CC [6–8]. Despite this, only a small percentage of cervical squamous cell carcinoma and endocervical adenocarcinoma (CESC) patients respond to immunotherapy [8]. Immune response participates in body stress caused by adverse conditions [9–11]. Therefore, it is urgent to explore new prognostic and therapeutic immunotherapy targets for CC.

A study found the role of apoptosis in oncology [12]. In recent years, abiotic stress can cause apoptosis [13,14]. In multicellular organisms, apoptosis is a common cell death process that acts as a natural barrier and protection against cancer development [15]. Furthermore, chemotherapeutic drugs can inhibit tumor growth via apoptosis [16]. Unfortunately, the dysregulation of tumor cell apoptosis mechanism can lead to tumorigenesis and drug resistance, which further causes treatment failure [17]. Aside from apoptosis, there were other novel cell death processes that have also been uncovered in recent years, such as pyroptosis, ferroptosis, entotic cell death, and necroptosis [12,18]. Among these, a relatively new programmed cell death process, necroptosis, provides a new cue for reversing apoptosis resistance [15].

Necrosis is a passive form of cell death. One study has discovered a novel cell death pattern that exhibits similar functional and morphological characteristics to necrosis, called necroptosis [19]. The classical apoptotic process is initiated by the activation of caspases [20]. Therefore, the apoptosis pathway can be inhibited due to the lack of caspases; subsequently, necroptosis is activated in this case instead of apoptosis [20]. In recent years, some scientists argued that apoptosis-resistant tumor cells may be sensitive to necroptosis [21,22].

Necroptotic cells release numerous pro-inflammatory molecules after plasma membrane permeabilization [15]. Similar to apoptosis, the core necroptosis signaling pathway is comprised of mixed lineage kinase domain-like proteins (MLKL/pMLKL) and receptor-interacting serine/threonine protein kinase 1/3 (RIPK1/RIPK3) [20]. A study found that the downregulation of MLKL/pMLKL and RIPK1/RIPK3 allow cancer cells to survive by evading necroptosis [23]. Meanwhile, necroptosis can suppress tumor progression by stimulating an important adaptive immune response and creating an immunosuppressive tumor microenvironment [24,25]. Therefore, necroptosis and its regulatory mechanisms may be important therapeutic targets for the tumor immunotherapy of CC. However, only a few necroptosis-related prognostic signatures for cancer were established.

In this study, we wanted to investigate the relationship between the expression levels of various necroptosis-related genes (NRGs) and the prognosis of CC patients and to establish a novel risk-score NRGs model for predicting the disease prognosis in CC. Validation experiment investigated the expression levels of the candidate NRGs in twenty-two CC tissues and matched adjacent normal tissues by IHC. Additionally, we aimed to validate the clinical value of the NRGs signature and its association with driver genes as well as the immune microenvironment in CC, which might provide new clues for the diagnosis and treatment of CC.

## Materials and methods

### Datasets and data processing

The UCSC Xena dataset (https://toil-xena-hub.s3.us-east-1.amazonaws.com/download/TcgaTargetGtex_rsem_gene_tpm.gz; Full metadata) was used to acquire TCGA and the Genotype-Tissue Expression (GTEx) expression and clinical information [26]. Dataset ID: TcgaTargetGtex_rsem_gene_tpm. Raw counts of RNA-sequencing data (level 3) and matching clinical data containing 306 cervical squamous cell carcinoma and endocervical adenocarcinoma (CESC) samples and 22 normal cervical samples (3 from TCGA, 19 from GTEX) were downloaded from TCGA and GTEx databases (Table 1). Two independent CC gene expression profiles (GSE151666 and GSE206224) were downloaded from the Gene Expression Omnibus (GEO) database (https://www.ncbi.nlm.nih.gov/geo/) and processed for analysis [27]. 159 NRGs were retrieved from Kyoto Encyclopedia of Genes and Genomes (KEGG) database [28]. All analytical methods were carried out utilizing the R software version v4.0.3. The expression data were normalized to transcripts per kilobase million (TPM) values before further analysis. Normalization of the read count values was performed using edger (R package).

**Table 1:** Clinical characteristics of patients with CESC

Characteristic	Levels	Overall
n		304
T stage, *n* (%)	T1	140 (58.1%)
	T2	71 (29.5%)
	T3	20 (8.3%)
	T4	10 (4.1%)
N stage, *n* (%)	N0	133 (68.9%)
	N1	60 (31.1%)
M stage, *n* (%)	M0	116 (92.1%)
	M1	10 (7.9%)
Clinical stage, *n* (%)	Stage I	162 (54.5%)
	Stage II	69 (23.2%)
	Stage III	45 (15.2%)
	Stage IV	21 (7.1%)
Radiation therapy, *n* (%)	No	122 (40.1%)
	Yes	182 (59.9%)
Primary therapy outcome, *n* (%)	PD	22 (10.1%)
	SD	6 (2.8%)
	PR	8 (3.7%)
	CR	181 (83.4%)
Race, *n* (%)	Asian	20 (7.7%)
	Black or African American	30 (11.6%)
	White	209 (80.7%)
Age, *n* (%)	≤ 50	186 (61.2%)
	> 50	118 (38.8%)
Weight, *n* (%)	≤ 70	138 (50.2%)
	> 70	137 (49.8%)
Height, *n* (%)	≤ 160	133 (51%)
	> 160	128 (49%)
BMI, *n* (%)	≤ 25	100 (38.6%)
	> 25	159 (61.4%)
Histological type, *n* (%)	Adenosquamous	52 (17.1%)
	Squamous cell carcinoma	252 (82.9%)
Histologic grade, *n* (%)	G1	18 (6.6%)
	G2	135 (49.6%)
	G3	118 (43.4%)
	G4	1 (0.4%)
Menopause status, *n* (%)	Pre	124 (53.7%)
	Peri	25 (10.8%)
	Post	82 (35.5%)
Birth control pill history, *n* (%)	No	89 (56.7%)
	Yes	68 (43.3%)
Keratinizing squamous cell carcinoma present, *n* (%)	No	119 (39.1%)
	Yes	185 (60.9%)
OS event, *n* (%)	Alive	233 (76.6%)
	Dead	71 (23.4%)
DSS event, *n* (%)	Alive	246 (82%)
	Dead	54 (18%)
PFI event, *n* (%)	Alive	233 (76.6%)
	Dead	71 (23.4%)
Age, median (IQR)		46 (38, 56.25)

*Abbreviations: CESC* cervical squamous cell carcinoma and endocervical adenocarcinoma, *PD* progressive disease, *CR* complete response, *SD* stable disease, *PR* partial response, *BMI* body mass index

### Tissue samples and immunohistochemical staining

Twenty-two cervical cancer tissues and paired normal tissues were obtained from Liuzhou People’s Hospital (China). A total of twenty-two patients had undergone surgery and received chemoradiotherapy or induction chemotherapy. Table S1 shows relevant clinical data. Before the study, we obtained informed consent from all patients and the study was approved by the Ethics Committee of Liuzhou People’s Hospital (Reference No. KY2022-035–01) and was performed according to the Declaration of Helsinki. All tissues were pathologically examined by three pathologists. Serial Sects. (4 μm) were cut from paraffin-embedded cervical cancer tissues. All sections were processed by dewaxing, hydration, removal of endogenous enzymes, and antigen repair. Subsequently, the slides were analyzed by immunohistochemistry with CAMK2A antibody (1:500; Sino Biological, China), CYBB (1:100; Proteintech, China), IL1A (1:100; Sangon Biotech, China), IL1B (1:200; Sino Biological, China), SLC25A5 (1:150; Proteintech, China), and TICAM2 (1:100; Sangon Biotech, China) as well as horseradish peroxidase-conjugated secondary antibodies (Maxim, China). To determine the expression of CAMK2A, CYBB, IL1A, IL1B, and SLC25A5, we calculated the mean of integrated optical density (IOD) for each slice using the Image-Pro Plus6.0 software (Media Cybernetics, USA).

### Differential expression analysis, mutation analysis, survival analysis, and correlation analysis

We utilized R packages “DESeq2” and “limma” to analyze the differentially expressed NRGs between CESC tumor and cervical normal tissues; the cutoff criteria were adjusted |Log2-fold change |> 1 and *P*-values < 0.05. We draw the Venn diagram in turn. We utilized the R packages “complex Heatmap,” “volcano,” “boxplot,” “ggplot2,” “survival,” and “survminer” to perform the complex heatmap, volcano plot, boxplot, expression analysis, and survival curves of the comparison between tumor tissues and normal tissues [29]. The mutation oncoplot waterfall and frequency plot of 43 PRGs in CESC patients were produced by the R package “maftools.” Spearman’s correlation and Pearson correlation analysis were utilized to examine the link and quantitative variables, respectively.

### Functional enrichment analysis

We used the R package “ClusterProfiler [30]” to carry out Gene Ontology (GO) enrichment analyses, including the biological process (BP), cellular component, and molecular function (MF) categories as well as KEGG pathway of co-expression genes, and visualized by R package “ggplot2.”

### The establishment of necroptosis risk scoring prognosis signature

Cox regression analysis was performed to evaluate the prognostic significance of the NRGs. Kaplan–Meier survival curves were built and hazard ratio (HR) with 95% confidence interval (CI) was calculated by log-rank tests. According to this, six prognostic NRGs were selected for further analysis. By using LASSO Cox regression, we constructed a prognostic model basing on these six prognostic NRGs [31], TCGA patients with CESC were divided into two subgroups (high- and low-risk) in accordance with the median risk score, and we compared OS between the two subgroups using KM analysis. Time ROC analysis was employed to predict accuracy of the risk score and each gene. Following this, a nomogram to quantitatively predict 1-, 3-, and 5-year overall survival was conducted via the clinical characteristics [32]. *P*-value, HR, and 95% CI of each variable were visualized by a forest via R package “forestplot”.

### Tumor Immune Estimation Resource (TIMER) database and Tumor-Immune System Interaction Database (TISIDB) analysis

TIMER (https://cistrome.shinyapps.io/timer/) dataset comprise six tumor-infiltrating immune subsets [33,34]. The levels of six subgroups for 10,897 tumors were precalculated in 32 cancers from the TCGA. The database analyzed gene expression and tumor immune infiltration (B cells, CD4 + T cells, CD8 + T cells, dendritic cells, macrophages, and neutrophils) in a variety of cancers. We utilized the TIMER dataset to examine the mRNA expression of these six prognostic NRGs in patients with CESC. TISIDB7 is an online website for tumor-immune system interaction^[[[[[ [35]]]^. Subsequently, we employed TISIDB to explore these six prognostic NRGs expression levels and tumor-infiltrating lymphocytes (TILs) as well as chemokines in CESC.

### Immune cell infiltration, TMB, MSI, and immune checkpoint analysis

To produce accurate immune infiltration estimates, we investigated immune cell infiltration, MB, MSI, and immunological checkpoints of NRGs in CESC by utilizing R packages “immunedeconv,” “ggplot2,” “pheatmap,” and “ggstatsplot.”

### Statistical analysis

Log-rank test, such as fold-change (FC), HR, and *P-*values were used to analyze data. Correlation between particular variables was measured via Spearman’s correlation analysis or Pearson correlation analysis, with the *r* values to measure the relationship strength. *P*-value or log-rank *p*-value of < 0.05 was judged as having statistical significance.

## Results

### Identification of necroptosis-related genes in CESC patients

**Fig. 1:**
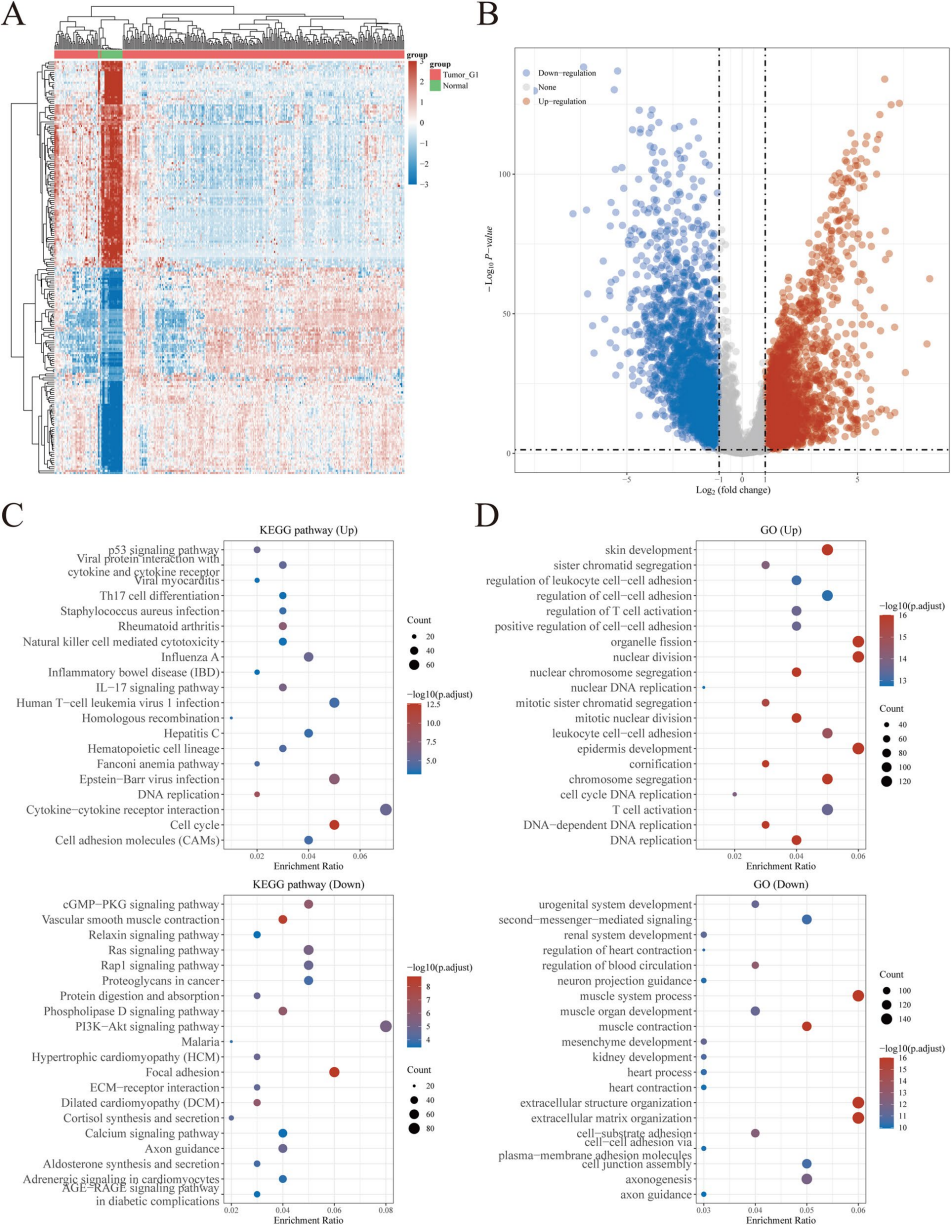
**A** Heatmap of the expression levels of 5492 DEGs in CESC. **B** Volcano plot of the expression levels of 5492 DEGs in CESC. **C**, **D** Enriched Gene Ontology terms and KEGG pathways associated with the 5492 DEGs in CESC. Abbreviations: DEGs, differentially expressed genes; CESC, cervical squamous cell carcinoma and endocervical adenocarcinoma; KEGG, Kyoto Encyclopedia of Genes and Genomes; GO, Gene Ontology

**Fig. 2:**
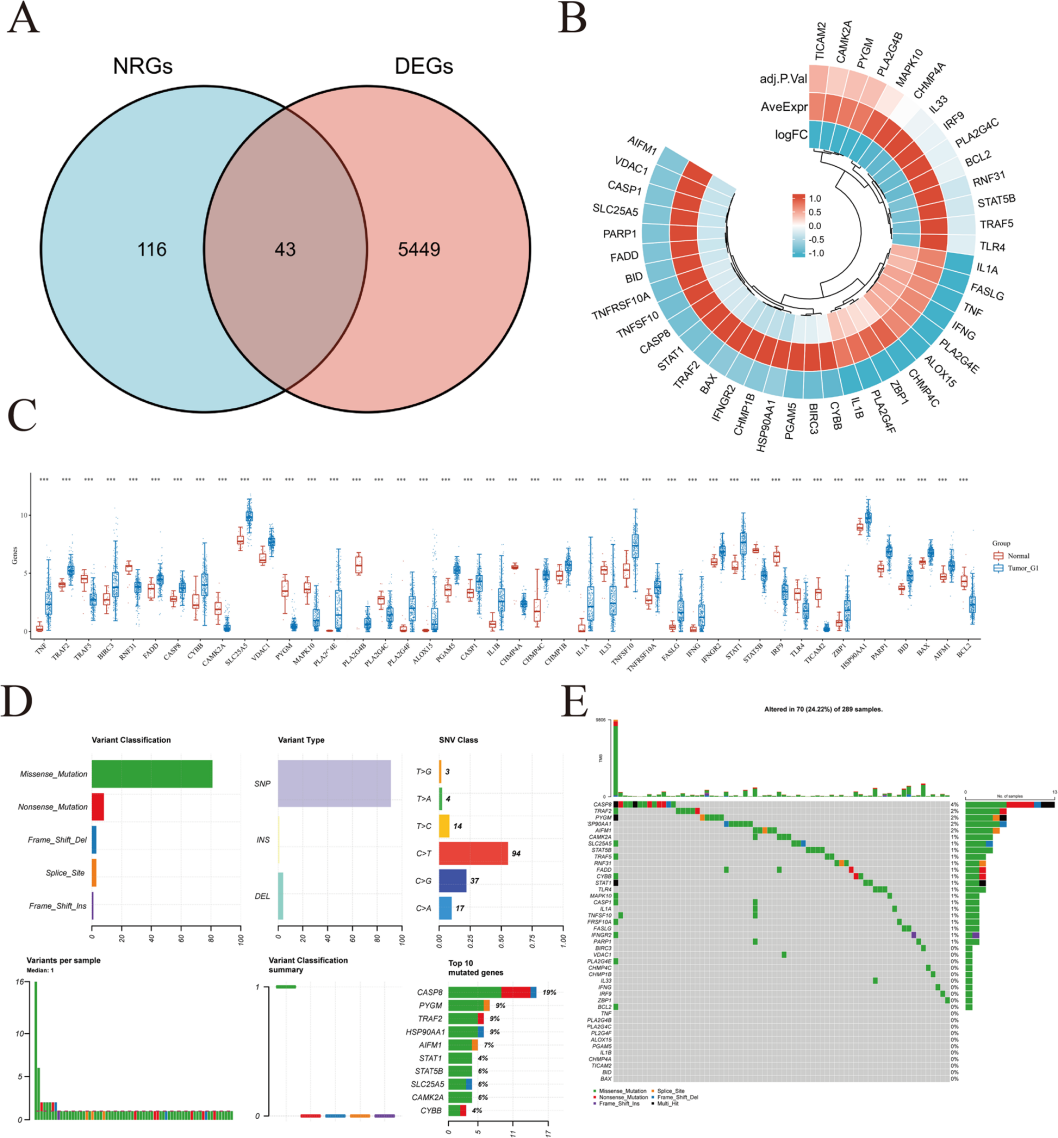
**A** Venn diagram of the intersection of NRGs and DEGs. **B** A total of 43 NRGs among the DEGs between CESC and normal samples. **C** The expression of 43 NRGs in CESC and normal bladder tissues, normal, red; tumor, blue. **D**, **E** The mutation frequency and classification of 43 NRGs in CESC. Abbreviations: CESC, cervical squamous cell carcinoma and endocervical adenocarcinoma; DEGs, differentially expressed genes; NRGs, necroptosis-related genes

We obtained the gene expression profiles, survival, and clinical data of 306 tumors and 22 normal samples of CESC patients from the TCGA and GTEx project databases. Patients with missing follow-up examination data were excluded from the samples. Next, we retrieved 159 NRGs from KEGG (Table S2). The cut-off criteria used to differentially expressed genes (DEGs) were |Log2-fold change |> 1 and adjusted *P*-values < 0.05. Based on the compiled limma, edgeR, and DESeq2 analysis results, we were able to identify 5492 DEGs between the 306 TCGA-CESC samples and 22 normal cervical samples (Fig. 1). A heat map and volcano plot were constructed to visualize the expression level of each gene in 328 specimens (Fig. 1A, B). Furthermore, enrichment analysis was used to explore the biological functions of these genes in CESC (Fig. 1C, D). From our analyses, a total of 43 NRGs, 29 upregulated and 14 downregulated, among these DEGs between CESC and normal samples were chosen for downstream analyses (Fig. 2A–C). The incidence of somatic mutations present in the 43 identified NRGs was examined and summarized. Our findings showed that 70 of 289 (24.22%) CESC samples displayed genetic mutations (Fig. 2D, E). The most common variant classification was missense mutations (Fig. 2D). Meanwhile, the most common variant type was single nucleotide polymorphisms (SNPs), and C > T ranked as the top single-nucleotide variation (SNV) class. Furthermore, our results showed that CAPS8, PYGM, TRAF2, HSP90AA1, AIFM1, STAT1, STAT5B, SLC25A5, CAMK2A, and CYBB were the top 10 genes among the 43 identified NRGs with the highest mutation frequency (Fig. 2E).

### Functional enrichment analysis

**Fig. 3:**
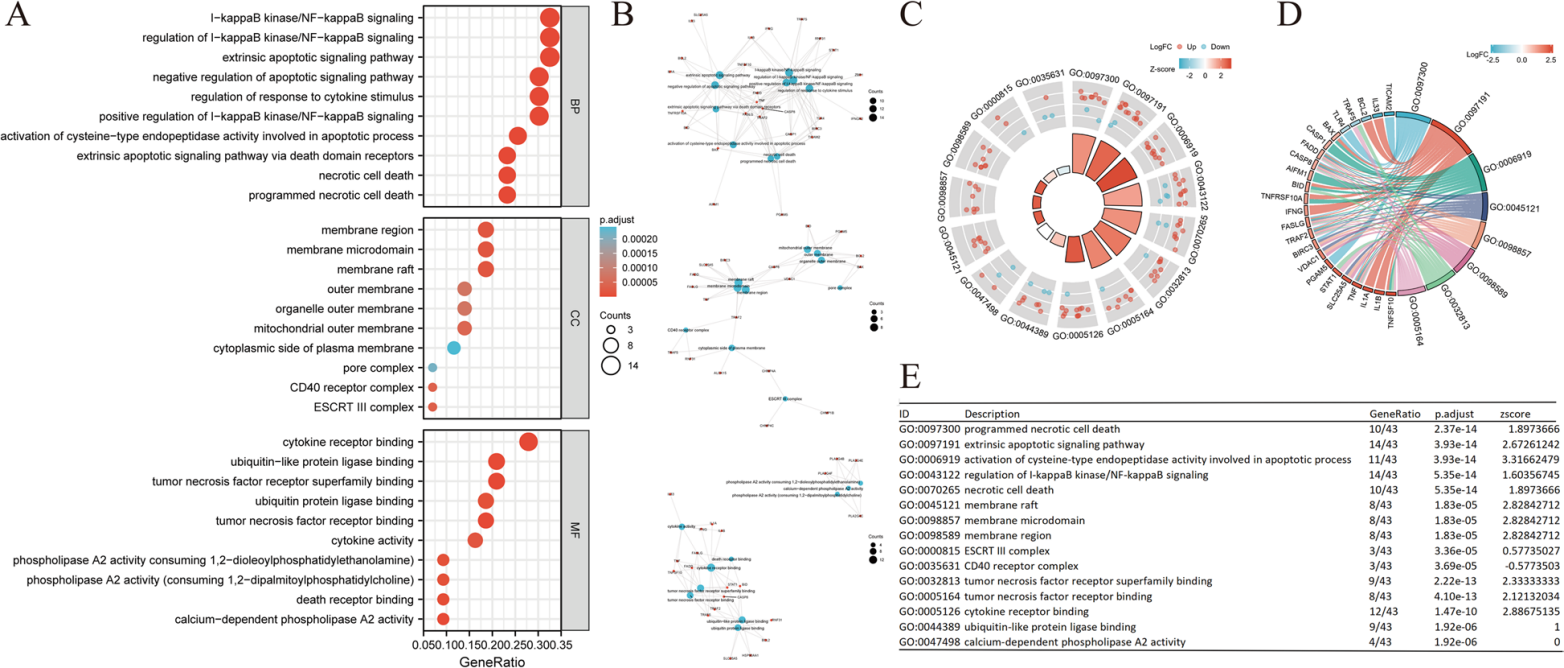
GO analysis of differentially expressed NRGs in CESC. **A** The significant terms of GO function enrichment. **B** Network diagram, blue nodes represent items, red nodes represent molecules, and the lines represent the relationship between items and molecules. **C** GO circle shows scatter map of the specified gene’s logFC. **D** Enrichment string diagrams of NRGs. **E** Enrichment analysis network diagram, description of pathways. Abbreviations: CESC, cervical squamous cell carcinoma and endocervical adenocarcinoma; GO, Gene Ontology; BP, biological process; CC cellular component; MF, molecular function; NRGs, necroptosis-related genes

**Fig. 4:**
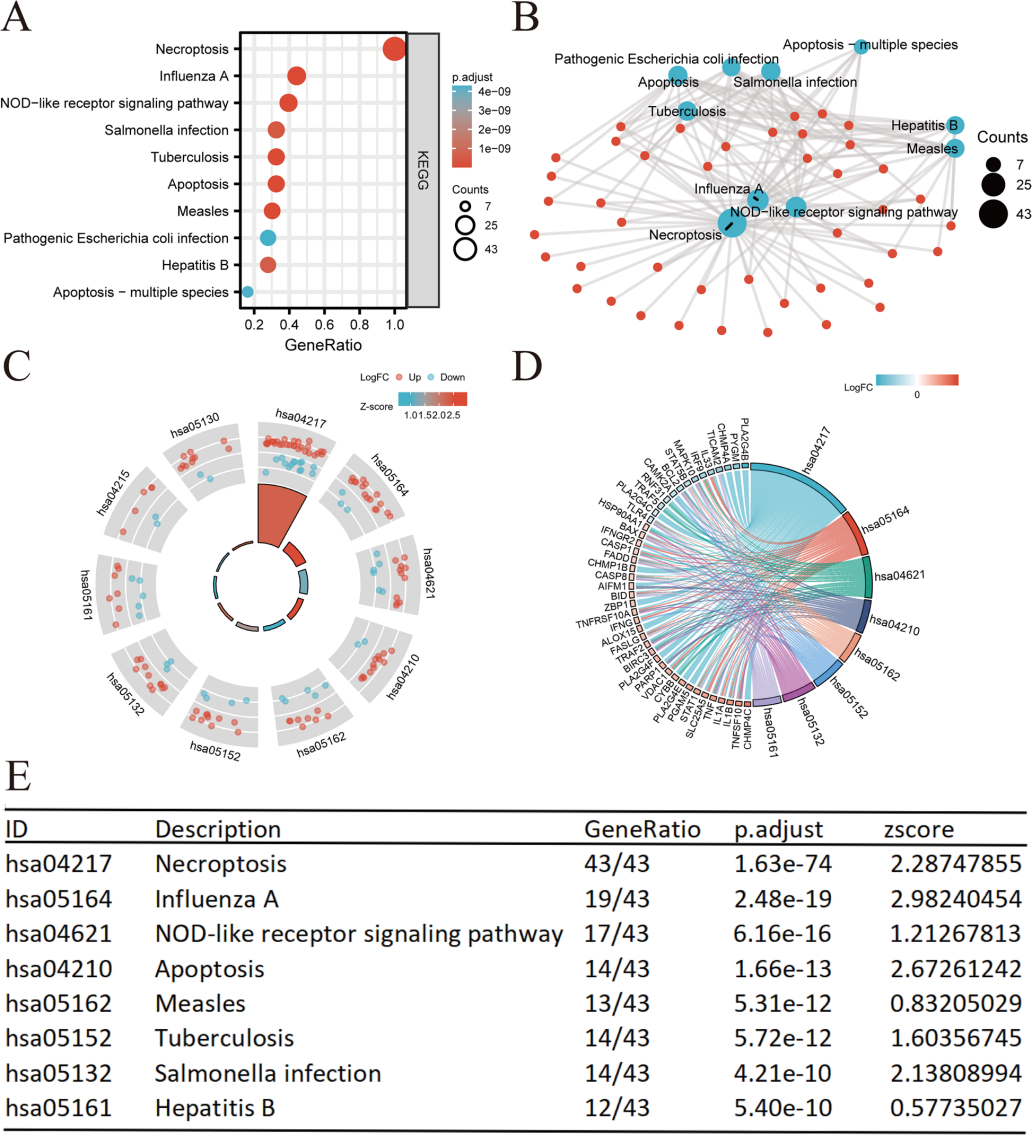
KEGG analysis of differentially expressed NRGs in CESC. **A** The significant terms of KEGG analysis. **B** Network diagram, blue nodes represent items, red nodes represent molecules, and the lines represent the relationship between items and molecules **C** KEGG circle shows scatter map of the specified gene’s logFC. **D** Enrichment string diagrams of NRGs. **E** Enrichment analysis network diagram, description of pathways. Abbreviations: CESC, cervical squamous cell carcinoma and endocervical adenocarcinoma; KEGG, Kyoto Encyclopedia of Genes and Genomes; NRGs, necroptosis-related genes

Functional enrichment analysis was performed to determine the biological characteristics of the 43 identified NRGs (Table S3). GO term enrichment and KEGG pathway analysis results are summarized in Figs. 3 and 4. The topmost enriched GO terms for biological processes (BP) included I − kappaB kinase/NF − kappaB signaling, regulation of I − kappaB kinase/NF − kappaB signaling, extrinsic apoptotic signaling pathway, negative regulation of apoptotic signaling pathway, and regulation of response to cytokine stimulus. The cellular components of these genes included membrane region, membrane microdomain, membrane raft, outer membrane, and organelle outer membrane. In terms of molecular function (MF), the terms cytokine receptor binding, ubiquitin − like protein ligase binding, tumor necrosis factor (TNF) receptor superfamily binding, ubiquitin protein ligase binding, and TNF receptor binding were enriched (Fig. 3A, B). KEGG pathway enrichment analysis showed that the 43 NRGs were correlated with pathways such as necroptosis, influenza A, NOD − like receptor signaling pathway, salmonella infection, and tuberculosis (Fig. 4A, B). Lastly, we combined our results with *z*-scores to predict the specific functions of the 43 NRGs in these pathways (Figs. 3C–E and 4C–E).

### The construction and validation of prognostic value of the risk scoring model

**Fig. 5:**
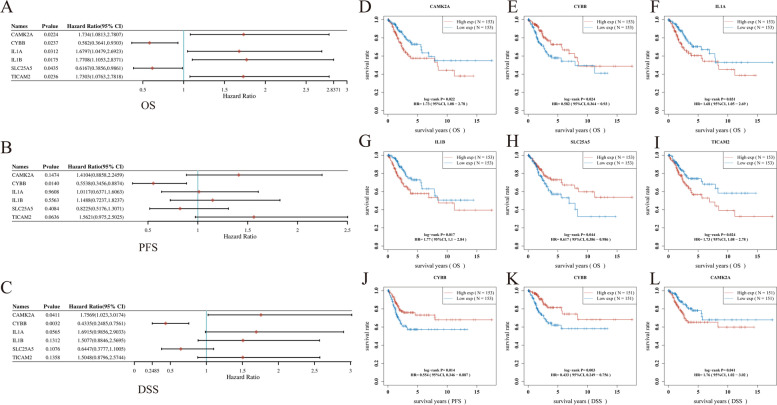
Prognostic analysis of six NRGs mRNA expression levels in patients with CESC in the TCGA database (**A**–**L**). *p < 0.05, **p < 0.01, ***p < 0.001. Abbreviations: NRGs, necroptosis-related gene; CESC, cervical squamous cell carcinoma and endocervical adenocarcinoma; OS, overall survival; PFS, progress-free survival; DSS, disease-specific survival

Univariate Cox regression analysis was employed to identify six NRGs, CAMK2A, CYBB, IL1A, IL1B, SLC25A5, and TICAM2, and determine their association with the prognosis of CESC patients (Table S4, Fig. 5A–C). The results showed that elevated mRNA expression levels of CAMK2A, IL1A, IL1B, and TICAM2 were correlated with worse OS of CESC patients (HR = 1.73, *P* = 0.022; HR = 1.68, *P* = 0.0031; HR = 1.77, *P* = 0.017; HR = 1.73, *P* = 0.024; Fig. 5D, F, G, and I). On the contrary, higher expression levels of CYBB and SLC25A5 were associated with improved disease prognosis (HR = 0.582, *P* = 0.024; HR = 0.617, *P* = 0.044; Fig. 5E, H). Specifically, higher CYBB expression values had substantial relationship with better progression-free survival (PFS; HR = 0.554, *P* = 0.014) and disease-specific survival (DSS) rates (HR = 0.433, *P* = 0.003) in CESC patients (Fig. 5J, K). In contrast, the expression level of CAMK2A was significantly correlated with worse DSS in CESC patients (HR = 1.76, *P* = 0.041; Fig. 5K).

**Fig. 6:**
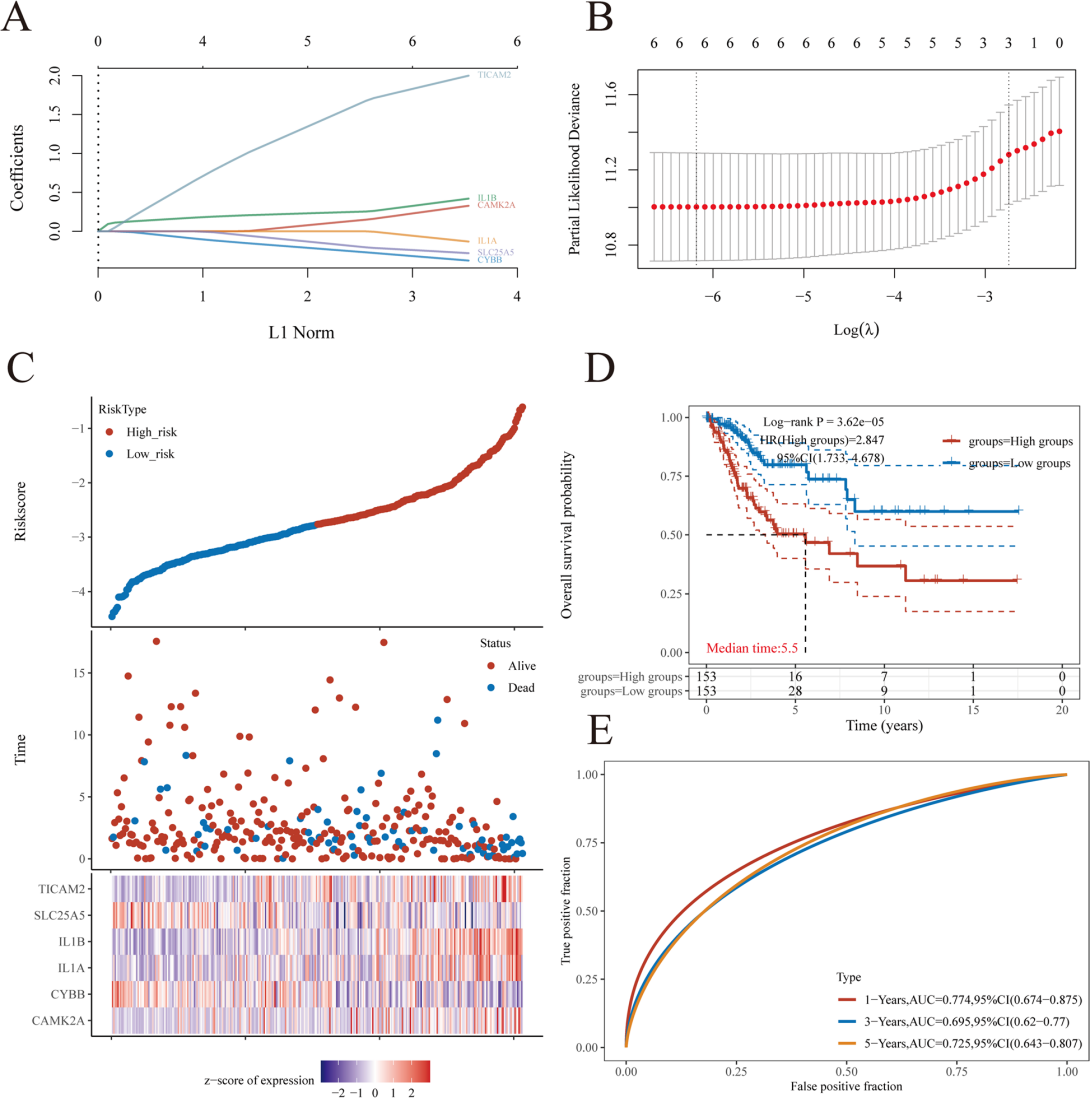
Establishment of a prognostic NRGs model. **A** LASSO coefficient profiles of six NRGs. **B** Plots of the tenfold cross-validation error rates. **C** Distribution of risk score, survival status, and the expression of six prognostic NRGs in NRGs. **D** Overall survival curves for CESC patients in the high-/low-risk group. **E** The ROC curve of measuring the predictive value. *p < 0.05, **p < 0.01, ***p < 0.001. Abbreviations: NRGs, necroptosis-related gene; CESC, cervical squamous cell carcinoma and endocervical adenocarcinoma; LASSO, least absolute shrinkage and selection operator; ROC, receiver operating characteristic

Incorporating the abovementioned findings with the results of the LASSO regression analysis, the corresponding six genes were selected for the construction of the gene signature. The model had the best fit to the data, while the penalty coefficient was found to be six (Fig. 6A, B). Subsequently, we performed a multivariate Cox regression analysis on the six NRGs. The results showed that the six NRGs could be used as prognostic predictors when coupled with the beta value of the multivariate Cox regression analysis. The risk score was calculated using the following formula: risk score = (0.3173) × CAMK2A + (− 0.3688) × CYBB + (− 0.123) × IL1A + (0.4098) × IL1B + (− 0.2759) × SLC25A5 + (1.9803) × TICAM2. The risk score for each patient was estimated using the formula. Then, the patients were grouped into either the high-risk or low-risk groups based on the median value of the risk score. The expression levels of the six genes, risk score distribution, and survival status were summarized in Fig. 6C. While the risk score increased, the patients’ death risk increased while the survival time decreased (Fig. 6D). Results showed that CESC patients with high-risk scores had worse OS than those with low-risk scores (HR = 2.847, Log *p* = 3.62e − 05), and this signature was validated by the time-dependent ROC curve (Fig. 6D, E). The area under the curves (AUCs) for the 1-, 3-, and 5-year OS of CESC patients were 0.774, 0.695, and 0.725, respectively, which revealed high accuracy of the constructed gene signature model (Fig. 6E).

### Building a predictive nomogram

**Fig. 7:**
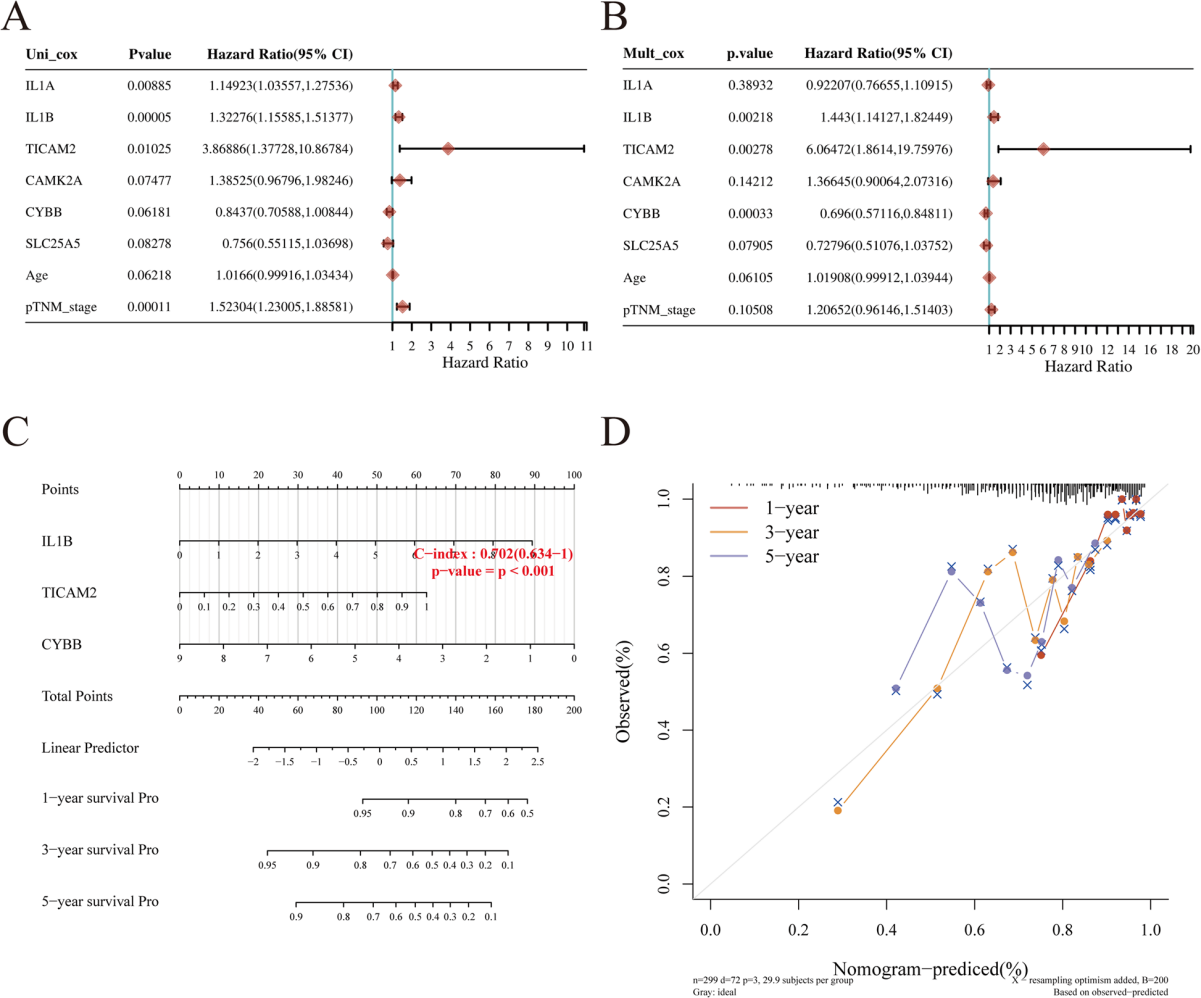
Construction of a predictive nomogram. **A**, **B** Hazard ratio and *P*-value of constituents involved in univariate and multivariate Cox regression and some parameters of six prognostic NRGs in CESC. **C** Nomogram to predict the 1-year, 3-year, and 5-year overall survival rate of CESC patients. **D** Calibration curve for the overall survival nomogram model in the discovery group. A dashed diagonal line represents the ideal nomogram. Abbreviations: NRGs, necroptosis-related gene; CESC, cervical squamous cell carcinoma and endocervical adenocarcinoma. *p < 0.05, **p < 0.01, ***p < 0.001

A nomogram was constructed to predict the survival probability of CESC patients by combining the gene expression data of the six prognostic NRGs and available clinicopathologic features. Univariate and multivariate analysis findings showed that the expression of IL1B and TICAM2 were independent factors that affected the prognosis of CESC patients (Fig. 7A, B). Furthermore, univariate analyses found that IL1A expression and pTNM-stage were independent factors affecting CESC prognosis. Meanwhile, CYBB expression was found to be an independent factor affecting CESC prognosis by multivariate analyses (Fig. 7A, B). Based on our findings, IL1B, TICAM2, and CYBB expression levels and pTNM-stage data were integrated into the nomogram model (C-index: 0.702, *p* < 0.001; Fig. 7C). The predictive nomogram indicated that the 1-, 3-, and 5-year OS rates could be predicted relatively well compared with an ideal model using the entire cohort (Fig. 7C, D).

### Relationship between the expression levels of the six NRGs and clinical characteristics of CESC patients

Since the expression levels of the six prognostic NRGs play significantly different prognostic roles in CESC, we explored the relationship of their expression levels with the different clinical and molecular criteria in CESC via the TCGA database. For the criterion of tumor pathologic stage, our analyses showed that IL1B was downregulated in patients with stage I and II CESC compared with patients in stages III and IV (*P* < 0.05; Table 5). Meanwhile, for the criterion of T stage, IL1B and TICAM2 had lower expression levels in patients with T1 and T2 CESC than those in the T3 and T4 group (*P* < 0.01; Table 5, 7). Notably, for histological type, CAMK2A, CYBB, IL1A, IL1B, and SLC25A5 were found to be upregulated in patients with cervical squamous cell carcinoma compared with those having cervical adenosquamous carcinoma (*P* < 0.05; Tables 2, 3, 4, 5, and 6). Furthermore, based on primary therapy outcome, the expression levels of IL1A, IL1B, and TICAM2 were significantly higher in progressive disease (PD) patients with CESC than those in the stable disease (SD), partial response (PR), and complete response (CR) patients (*P* < 0.05; Tables 4, 5, and 7). The expression levels of CAMK2A and IL1A were also found to be associated with the menopause status of CESC patients (*P* < 0.05; Tables 2 and 3). Additionally, IL1A expression was higher in CESC patients aged ≤ 50 years than patients in the > 50 age group (*P* < 0.05; Table 2). Moreover, CESC patients with a body mass index (BMI) ≤ 25 had significantly higher expression levels of CAMK2A than patients with a BMI > 25 (*P* < 0.05; Table 2). Overall, these findings showed that the expression levels of the six prognostic NRGs were associated with tumor stage, T stage, histological type, and therapy outcome of CESC patients.

**Table 2:** Relationship between of CAMK2A expression and clinical characteristics of patients with CESC

Gene	Characteristics	Total (*N*)	Odds ratio (OR)	*P* value
CAMK2A	Age (≤ 50 vs. > 50)	304	1.657 (0.992–2.910)	0.064
	BMI (≤ 25 vs. > 25)	259	2.047 (1.196–3.624)	0.011
	Menopause status (Pre vs. Peri and Post)	231	1.885 (1.070–3.519)	0.035
	Histological type (adenosquamous vs. squamous cell carcinoma)	304	0.008 (0.001–0.057)	< 0.001
	T stage (T1 and T2 vs. T3 and T4)	241	0.692 (0.356–1.475)	0.301
	N stage (N0 vs. N1)	193	0.914 (0.496–1.753)	0.777
	M stage (M0 vs. M1)	126	1.711 (0.441–14.791)	0.534
	Clinical stage (stage I and stage II vs. stage IV and stage III)	297	0.873 (0.512–1.558)	0.629
	Histologic grade (G1 and G2 vs. G4 and G3)	272	1.382 (0.810–2.441)	0.246
	Primary therapy outcome (SD and PR and CR vs. PD)	217	0.792 (0.347–2.149)	0.607
	Radiation therapy (yes vs. no)	304	0.803 (0.497–1.298)	0.366

*Abbreviations: CESC* cervical squamous cell carcinoma and endocervical adenocarcinoma, *PD*, progressive disease, *CR* complete response, *SD* stable disease, *PR* partial response, *BMI* body mass index

**Table 3:** Relationship between of CYBB expression and clinical characteristics of patients with CESC

Gene	Characteristics	Total (*N*)	Odds ratio (OR)	*P* value
CYBB	Age (≤ 50 vs. > 50)	304	0.865 (0.723–1.030)	0.106
	BMI (≤ 25 vs. > 25)	259	0.970 (0.801–1.172)	0.750
	Menopause status (Pre vs. Peri and Post)	231	0.956 (0.783–1.166)	0.654
	Histological type (adenosquamous vs. squamous cell carcinoma)	304	0.541 (0.411–0.699)	< 0.001
	T stage (T1 and T2 vs. T3 and T4)	241	1.098 (0.823–1.474)	0.526
	N stage (N0 vs. N1)	193	0.936 (0.741–1.180)	0.576
	M stage (M0 vs. M1)	126	1.649 (1.000–2.817)	0.055
	Clinical stage (stage I and stage II vs. stage IV and stage III)	297	1.072 (0.873–1.321)	0.509
	Histologic grade (G1 and G2 vs. G4 and G3)	272	0.843 (0.702–1.009)	0.065
	Primary therapy outcome (SD and PR and CR vs. PD)	217	1.397 (0.997–1.991)	0.057
	Radiation therapy (yes vs. no)	304	1.074 (0.902–1.280)	0.424

*Abbreviations: CESC* cervical squamous cell carcinoma and endocervical adenocarcinoma, *PD* progressive disease, *CR* complete response, *SD* stable disease, *PR* partial response, *BMI* body mass index

**Table 4:** Relationship between of IL1A expression and clinical characteristics of patients with CESC

Gene	Characteristics	Total (*N*)	Odds ratio (OR)	*P* value
IL1A	Age (≤ 50 vs. > 50)	304	1.153 (1.029–1.298)	0.016
	BMI (≤ 25 vs. > 25)	259	1.076 (0.953–1.215)	0.238
	Menopause status (Pre vs. Peri and Post)	231	1.182 (1.040–1.351)	0.012
	Histological type (adenosquamous vs. squamous cell carcinoma)	304	0.234 (0.140–0.357)	< 0.001
	T stage (T1 and T2 vs. T3 and T4)	241	0.877 (0.738–1.047)	0.138
	N stage (N0 vs. N1)	193	0.997 (0.861–1.158)	0.963
	M stage (M0 vs. M1)	126	1.004 (0.743–1.419)	0.980
	Clinical stage (Stage I and stage II vs. stage IV and stage III)	297	0.893 (0.787–1.015)	0.080
	Histologic grade (G1 and G2 vs. G4 and G3)	272	1.088 (0.967–1.227)	0.164
	Primary therapy outcome (SD and PR and CR vs. PD)	217	0.819 (0.675–0.995)	0.042
	Radiation therapy (yes vs. no)	304	0.988 (0.886–1.103)	0.827

*Abbreviations: CESC* cervical squamous cell carcinoma and endocervical adenocarcinoma, *PD* progressive disease, *CR* complete response, *SD* stable disease, *PR* partial response, *BMI* body mass index

**Table 5:** Relationship between of IL1B expression and clinical characteristics of patients with CESC

Gene	Characteristics	Total (*N*)	Odds ratio (OR)	*P* value
IL1B	Age (≤ 50 vs. > 50)	304	1.043 (0.902–1.209)	0.573
	BMI (≤ 25 vs. > 25)	259	1.136 (0.966–1.339)	0.124
	Menopause status (Pre vs. Peri and Post)	231	1.121 (0.949–1.331)	0.183
	Histological type (adenosquamous vs. squamous cell carcinoma)	304	0.489 (0.361–0.640)	< 0.001
	T stage (T1 and T2 vs. T3 and T4)	241	0.719 (0.572–0.900)	0.004
	N stage (N0 vs. N1)	193	1.045 (0.857–1.284)	0.668
	M stage (M0 vs. M1)	126	1.073 (0.715–1.731)	0.751
	Clinical stage (stage I and stage II vs. stage IV and stage III)	297	0.846 (0.717–1.000)	0.048
	Histologic grade (G1 and G2 vs. G4 and G3)	272	0.892 (0.763–1.041)	0.149
	Primary therapy outcome (SD and PR and CR vs. PD)	217	0.758 (0.588–0.980)	0.031
	Radiation therapy (yes vs. no)	304	0.977 (0.847–1.130)	0.756

*Abbreviations: CESC* cervical squamous cell carcinoma and endocervical adenocarcinoma, *PD* progressive disease, *CR* complete response, *SD* stable disease, *PR* partial response, *BMI* body mass index

**Table 6:** Relationship between of SLC25A5 expression and clinical characteristics of patients with CESC

Gene	Characteristics	Total (*N*)	Odds ratio (OR)	*P* value
SLC25A5	Age (≤ 50 vs. > 50)	304	0.802 (0.574–1.109)	0.188
	BMI (≤ 25 vs. > 25)	259	1.008 (0.716–1.425)	0.963
	Menopause status (Pre vs. Peri and Post)	231	0.900 (0.622–1.295)	0.573
	Histological type (adenosquamous vs. squamous cell carcinoma)	304	0.648 (0.430–0.975)	0.037
	T stage (T1 and T2 vs. T3 and T4)	241	0.576 (0.327–0.985)	0.050
	N stage (N0 vs. N1)	193	1.129 (0.763–1.670)	0.540
	M stage (M0 vs. M1)	126	1.515 (0.689–3.164)	0.278
	Clinical stage (stage I and stage II vs. stage IV and stage III)	297	0.926 (0.627–1.353)	0.694
	Histologic grade (G1 and G2 vs. G4 and G3)	272	0.944 (0.680–1.308)	0.731
	Primary therapy outcome (SD and PR and CR vs. PD)	217	1.688 (0.918–3.093)	0.089
	Radiation therapy (yes vs. no)	304	1.028 (0.745–1.417)	0.865

*Abbreviations: CESC* cervical squamous cell carcinoma and endocervical adenocarcinoma, *PD* progressive disease, *CR* complete response, *SD* stable disease, *PR* partial response, *BMI* body mass index

**Table 7:** Relationship between of TICAM2 expression and clinical characteristics of patients with CESC

Gene	Characteristics	Total (*N*)	Odds ratio (OR)	*P* value
TICAM2	Age (≤ 50 vs. > 50)	304	0.447 (0.123–1.621)	0.219
	BMI (≤ 25 vs. > 25)	259	1.924 (0.440–8.369)	0.381
	Menopause status (Pre vs. Peri and Post)	231	0.590 (0.131–2.619)	0.488
	Histological type (adenosquamous vs. squamous cell carcinoma)	304	0.144 (0.018–0.922)	0.053
	T stage (T1 and T2 vs. T3 and T4)	241	0.074 (0.010–0.536)	0.009
	N stage (N0 vs. N1)	193	0.624 (0.101–4.073)	0.614
	M stage (M0 vs. M1)	126	0.454 (0.012–33.647)	0.690
	Clinical stage (stage I and stage II vs. stage IV and stage III)	297	0.278 (0.063–1.258)	0.091
	Histologic grade (G1 and G2 vs. G4 and G3)	272	3.791 (0.924–16.721)	0.070
	Primary therapy outcome (SD and PR and CR vs. PD)	217	0.083 (0.009–0.783)	0.025
	Radiation therapy (yes vs. no)	304	1.287 (0.356–4.799)	0.702

*Abbreviations: CESC* Cervical squamous cell carcinoma and endocervical adenocarcinoma, *PD* progressive disease, *CR* complete response, *SD* stable disease, *PR* partial response, *BMI* body mass index

### Protein expression analysis of CAMK2A, CYBB, IL1A, IL1B, SLC25A5, and TICAM2 in CESC

**Fig. 8:**
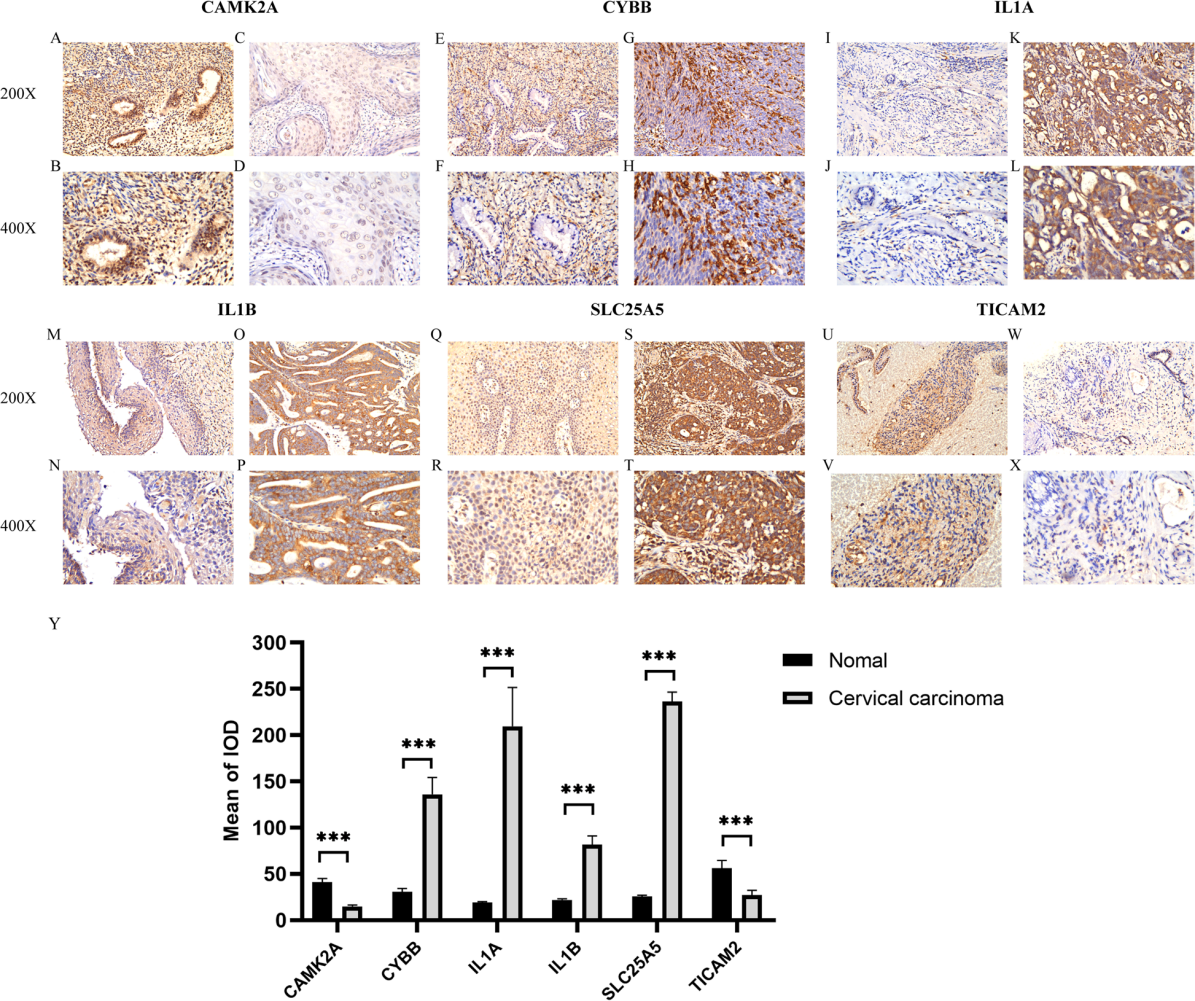
Six NGRs protein expression analysis. **A**, **E**, **I**, **M**, **Q**,** U** CAMK2A, CYBB, IL1A, IL1B, SLC25A5, and TICAM2 protein expression in normal cervical tissue (200 × magnification). **B**, **F**, **J**, **N**, **R**, **V** CAMK2A, CYBB, IL1A, IL1B, SLC25A5, and TICAM2 protein expression in normal cervical tissue (400 × magnification). **C**, **G**, **K**, **O**, **S**, **W** CAMK2A, CYBB, IL1A, IL1B, SLC25A5, and TICAM2 protein expression in CESC tumor tissue (200 × magnification). **D**, **H**, **L**, **P**, **T**,** X** CAMK2A, CYBB, IL1A, IL1B, SLC25A5, and TICAM2 protein expression in CESC tumor tissue (400 × magnification). **Y** Quantification of immunostains for CAMK2A, CYBB, IL1A, IL1B, SLC25A5, and TICAM2 by IOD analysis. ***p < 0.001

The protein expression of the six NRGs were validated by IHC in twenty-two pairs of CESC tumor tissues and adjacent normal tissues. As revealed by IHC staining analysis, the proteins of CYBB, IL1A, IL1B, and SLC25A5 were mainly found in cancer cells’ nucleus, cytoplasm, and membranes; brown staining indicated positive staining (Fig. 8G, H, K, L,O, P, S, T); and these NRG proteins were either weakly expressed or not expressed in normal tissues (Fig. 8E, F, I, J, M, N, Q, R). On the contrary, protein expressions of CAMK2A and TICAM2 were located primarily in normal tissues (Fig. 8A, B, U, V) and weakly expressed in tumor tissues (Fig. 8C, D, W, X). Using value of IOD, quantification of immunohistochemical analysis showed that protein expressions of CYBB, IL1A, IL1B, and SLC25A5 were significantly higher in CESC tissues than in adjacent non-tumor tissues (*P* < 0.001) (Fig. 8Y). In striking contrast, CAMK2A and TICAM2 protein expression was found to be greater in normal tissues than in cancerous tissues (*P* < 0.001) (Fig. 8Y).

### The relationship between risk score of necroptosis risk scoring signature and expression levels of PIK3CA, PTEN, TP53, STK11, and KRAS

**Fig. 9:**
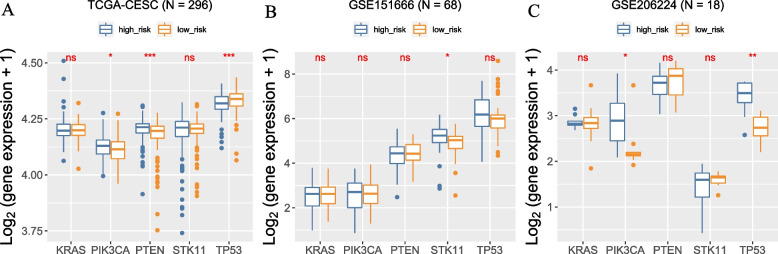
The relationship between risk score of necroptosis risk scoring signature and expression levels of PIK3CA, PTEN, TP53, STK11, and KRAS in TCGA and GEO databases. *p < 0.05, **p < 0.01, ***p < 0.001

Previous studies have implicated cervical cancer might be driven by cell cycle genes and antiviral genes [36]. Among the identified genes, TP53, PIK3CA, PTEN, STK11, and KRAS were previously shown to drive CC [36]. So, we next explored the relationship between risk score of our necroptosis risk scoring signature and the expression of these five driver genes. As shown in Fig. 9A, the expression levels of PIK3CA (*P* < 0.05) and PTEN (*P* < 0.001) were higher in CESC patients with high-risk scores than those with low-risk scores in TCGA database, while the expression levels of TP53 were lower in high-risk scores than low-risk scores. In GSE151666, only STK11 expression was higher in CESC patients with high-risk scores than that with low-risk scores(*P* < 0.05) (Fig. 9B). In GSE206224, PIK3CA (*P* < 0.05) and TP53 (*P* < 0.01) expression levels were higher in CESC patients with high-risk scores than low-risk scores (Fig. 9B).

### The correlations between expression levels of six NRGs and immune cell infiltration in CESC

**Fig. 10:**
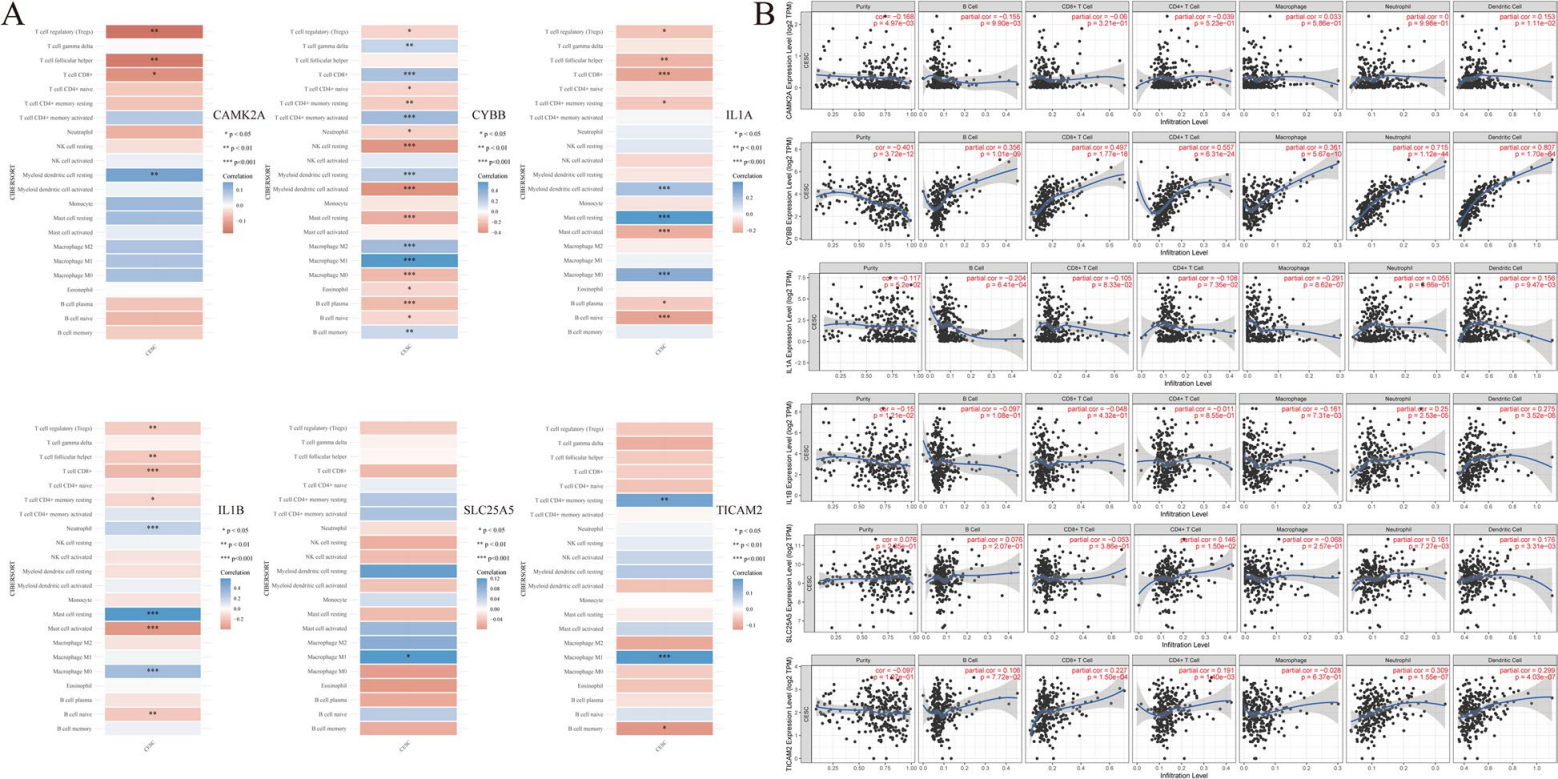
**A** the relationship between six prognostic NRGs expression levels and immune cell infiltration in CESC. **B** The relationship between six prognostic NRGs expression levels and immune cell infiltration in CESC via TIMER database. Abbreviations: NRGs, necroptosis-related gene; CESC, cervical squamous cell carcinoma and endocervical adenocarcinoma; TILs, tumor-infiltrating lymphocytes. *p < 0.05, **p < 0.01, ***p < 0.001

Several studies have shown that tumor-infiltrating lymphocytes (TILs) are independent predictors of tumor stage, grade, and lymph node status in cancers [37,38]. Based on the findings that the expression of the six NRGs was related to various clinical characteristics in CESC, we sought to investigate the association between the expression levels of the six prognostic NRGs and immune cell infiltration in CESC using the TCGA dataset. Our results showed that the mRNA expression of CAMK2A was positively associated with myeloid dendritic cell resting (*P* < 0.001) and was negatively correlated with T cell regulatory (Tregs), T cell follicular helper, and T cell CD8 + (*P* < 0.05; Fig. 10A). Meanwhile, CYBB expression level was significantly positively associated with T cell gamma, T cell CD8 + , T cell CD4 + memory activated, myeloid dendritic cell resting, macrophage M1, macrophage M2, and B cell memory cells (*P* < 0.01), and were negatively correlated with T cell regulatory (Tregs), T cell CD4 + naive, T cell CD4 + memory resting, neutrophil, NK cell resting, myeloid dendritic cell activated, mast cell resting, macrophage M0, eosinophil, B cell plasma, and B cell naive (*P* < 0.05; Fig. 8A). Furthermore, IL1A expression was significantly positively associated with myeloid dendritic cell activated, mast cell resting, and macrophage M0 (*P* < 0.001) and was significantly negatively associated with T cell regulatory (Tregs), T cell follicular helper, T cell CD8 + , T cell CD4 + memory resting, mast cell activated, B cell plasma, and B cell naive (*P* < 0.05; Fig. 10A). Notably, IL1B expression level was strongly positively associated with neutrophil, mast cell resting, and macrophage M0 (*P* < 0.001) and was significantly negatively associated with Tregs, T cell follicular helper, T cell CD8 + , T cell CD4 + memory resting, mast cell activated, and B cell naive (*P* < 0.05; Fig. 10A). TICAM2 expression was positively associated with T cell CD4 + memory resting and macrophage M1 (*P* < 0.01) and was negatively correlated with B cell memory (*P* < 0.05; Fig. 10A). Lastly, the expression level of SLC25A5 was only positively correlated with macrophage M0 (*P* < 0.05).

**Fig. 11:**
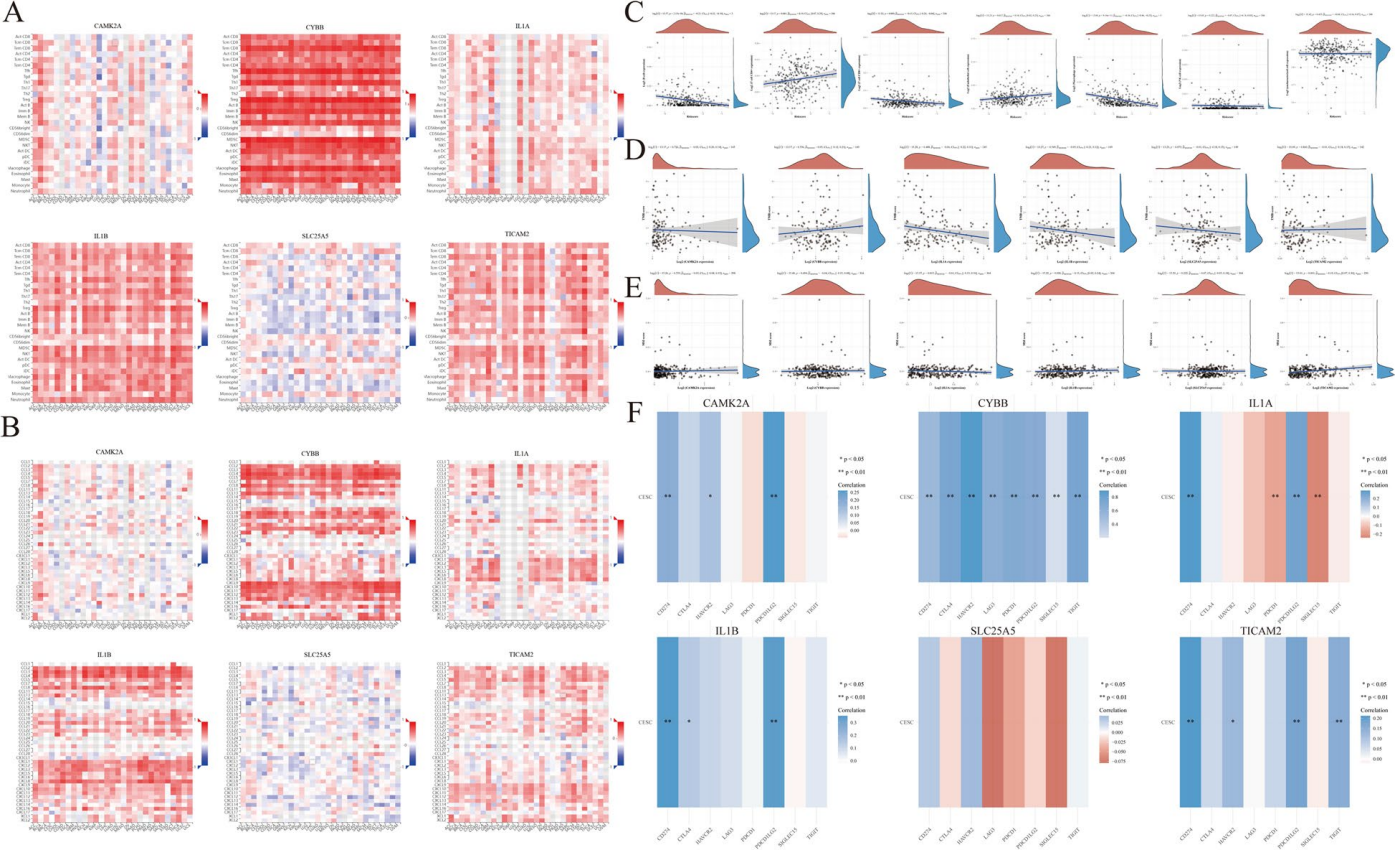
**A**, **B** Relations between the expression of six prognostic NRGs and 28 types of TILs and Chemokine across human Cancers via TISIDB online tool. **C** The relationship between six prognostic NRGs expression levels and score in CESC via TCGA database. **D** The correlation between six prognostic NRGs and TMB in CESC. **E** The correlation between six prognostic NRGs and MSI in CESC. **F** The relationship between six prognostic NRGs expression levels and immune checkpoints in CESC via TCGA database. *p < 0.05, **p < 0.01, ***p < 0.001. Abbreviations: NRGs, necroptosis-related gene; CESC, cervical squamous cell carcinoma and endocervical adenocarcinoma; TMB, tumor mutation burden; MSI, microsatellite instability

The TIMER database and TISIDB online tool were used to validate our findings (Figs. 10B and 11A). Our analyses using the TIMER2.0 online tools showed that the mRNA expression level of CAMK2A had a positive relationship with the levels of dendritic cells (DCs), and had a negative correlation with levels of B cells and tumor purity levels (*P* < 0.05; Fig. 10B). Meanwhile, the expression levels of CYBB were strongly positively associated with B cells, CD8 + T cells, CD4 + T cells, macrophages, neutrophils, and DCs (*P* < 0.001) and were only significantly negatively associated with tumor purity level (*P* < 0.001; Fig. 10B). On the other hand, expression levels of IL1A had a positive association with DCs (*P* < 0.05) and a negative correlation with B cells and macrophages (*P* < 0.001; Fig. 10B). The expression level of IL1B was significantly positively correlated with neutrophils and DCs (*P* < 0.001) and was significantly negatively associated with tumor purity level and macrophages (*P* < 0.05; Fig. 10B). SLC25A5 expression were positively correlated with CD4 + T cells, neutrophils, and DCs (*P* < 0.05; Fig. 10B). Additionally, TICAM2 expression levels were significantly positively associated with CD8 + T cells, CD4 + T cells, neutrophils, and DCs (*P* < 0. 01; Fig. 10B). Furthermore, we examined the relationship between the mRNA expression levels of the six NRGs and the levels of 28 types of TILs and chemokines using the TISIDB dataset. The results illustrated that CYBB and IL1B had a strong association with TILs and chemokines in CESC (Fig. 11A, B). Meanwhile, CAMK2A, IL1A, and TICAM2 showed a moderate correlation with TILs and chemokines (Fig. 11A, B). On the contrary, there was a weak correlation between SLC25A5 expression and TILs and chemokines in CESC (Fig. 11A, B). Taken together, these results demonstrated that most of the six NRGs had an instrumental function in the regulation of immune cell infiltration in CESC.

### Immune scores, TMB, MSI, and immune checkpoints analysis of NRGs

We explored the correlation between the risk scores from our model and immune scores. Our results show that the risk scores were positively correlated with T cell CD4 + (*p* = 0.001), endothelial cell (*p* = 0.014), and myeloid dendritic cell (*p* = 3.1e − 12) and were negatively correlated with B cell (*p* = 2.15e − 04), T cell CD8 + (*p* = 0.009), and macrophages (*p* = 9.16e − 11; Fig. 11C). Since TMB and MSI were suggested to be predictive biomarkers for cancer immunotherapy, we also analyzed the relationship between our NRGs gene signature, TMB, and MSI in CESC to explore whether the six NRGs could also be used as biomarkers for evaluating the efficacy of immunotherapy. Our results showed a positive relationship between MSI and either IL1B (*p* = 0.020) or TICAM2 (*p* = 0.001) in CESC patients (Fig. 11E). However, there was no significant correlation between TMB and any of the identified NRGs in CESC (*p* > 0.05; Fig. 11D).

SIGLEC15, PDCD1LG2(PD-L2), TIGIT, PDCD1(PD-1), CD274(PD-L1), CTLA4, LAG3, and HAVCR2(TIM3) are transcripts related to immunological checkpoints that perform vital functions in tumor immune evasion. Considering that the six identified NRGs in our model might be used as predictive biomarkers in CESC, we then explored the relationship between the NRGs and the various immunological checkpoints. Our findings showed that mRNA expression level of CYBB had strong positive correlations with SIGLEC15, PDCD1LG2, TIGIT, LAG3, CD274, CTLA4, HAVCR2, and PDCD1 expression in CESC (*P* < 0.01; Fig. 11F). Meanwhile, CAMK2A expression had positive correlations with SIGLEC15, TIGIT, LAG3, CD274, HAVCR2, and PDCD1LG2 expression (*P* < 0.05). Similarly, IL1B expression had positive relationships with CD274, CTLA4, and PDCD1LG2 expression in CESC (*P* < 0.05; Fig. 11F). Moreover, expression level of TICAM2 had significantly positive correlations with CD274, HAVCR2, PDCD1LG2, and TIGIT expression in CESC (*P* < 0.05; Fig. 11F). Lastly, expression level of IL1A was significantly positively correlated with CD274 and PDCD1LG2 (*P* < 0.01), while being negatively correlated with PDCD1 and SIGLEC15 (*P* < 0.01; Fig. 11F).

## Discussion

CC is a highly aggressive malignant tumor characterized by high incidence and recurrence rates worldwide [8]. The effectiveness of conventional treatments for advanced CC is limited [39]. Over the past few decades, advancements in molecular genotyping and phenotyping led to huge progress in targeted therapies and immunotherapy for CC [8,39,40]. Despite that, fatality rates of CESC remain high, and treatments are still restricted [41].

Necroptosis is a cellular process that is activated by extrinsic apoptotic receptors and is identified as a regulated form of necrosis [42]. As a highly inflammatory process, necroptosis can be activated under apoptosis-deficient conditions, which is similar to apoptosis under immune-suppressive conditions [43]. An early study indicated that the core signaling pathway of necroptosis participated in the process of tumorigenesis [44]. A recent study argued that necroptosis may overcome the apoptosis resistance of tumor cells and suppress the immune response against cancer [45]. However, the specific mechanism underlying tumor necroptosis remains poorly understood [46].

In this study, 43 differentially expressed NRGs, composed of 29 upregulated and 14 downregulated genes, were assessed between CESC and normal samples from the TCGA database. KEGG pathway and GO term functional enrichment analyses showed that these 43 NRGs were mostly involved in apoptotic processes, viral response, apoptotic mitochondrial changes, necrosis pathways, extrinsic apoptotic processes, Influenza A response, I − kappaB kinase/NF − kappaB, NOD − like receptor, and other signaling pathways. The cellular components of these genes were membrane region, membrane microdomain, membrane raft, outer membrane, and organelle outer membrane. In terms of molecular function, cytokine receptor binding, ubiquitin − like protein ligase binding, TNF receptor superfamily binding, ubiquitin protein ligase binding, and TNF receptor binding were enriched.

Next, we used the TCGA database to perform a survival analysis in relation to the expression of the 43 identified NRGs. Our findings showed that higher expression levels of CYBB and SLC25A5 might play as favorable prognostic biomarkers. On the contrary, elevated expression levels of CAMK2A, IL1A, IL1B, and TICAM2 may be used as unfavorable prognostic biomarkers in CESC patients. Some research argued that IL1A and IL1B were associated with tumor progression and prognosis; in addition, IL-1A was an independent predictor of survival in cervical cancer patients. [47–49]. Furthermore, IL1B could be predicted the risk of breast cancer patients developing bone metastases [50,51]. The results of our study were consistent with those findings. Although there have been few studies of the relationship between IL1A, IL1B, and tumorigenesis, almost no studies have been done to elucidate the expression and function of these six NRGs in CESC.

Since the expression levels of the six NRGs we identified were significantly different between tumor and normal tissues, we speculated that these NRGs may be used as a diagnostic tool for CESC. LASSO Cox regression analysis was used to construct a prognostic signature based on the six NRGs: CAMK2A, CYBB, IL1A, IL1B, SLC25A5, and TICAM2. Subsequently, we grouped the patients into either the high-risk or low-risk groups based on the median risk score value. Our findings revealed that the high-risk group had significantly lower OS than the low-risk group. Univariate and multivariate analyses showed that IL1B and TICAM2 were independent factors affecting the prognosis of CESC patients. Furthermore, univariate analyses found that IL1A expression and pTNM-stage were independent factors of CESC prognosis. Similarly, CYBB expression was found to be an independent factor affecting the prognosis of CECS through multivariate analyses (Fig. 7A, B). Our findings suggested that our prognostic signature may be used as an independent prognostic marker for CESC, which could predict the OS of CESC patients with medium-to-high accuracy. In addition, we constructed ROC curves to validate the prognostic signature as an independent indicator. Our findings showed that the 1-, 3-, and 5-year OS rates could be predicted by the model relatively well compared to an ideal model using the entire cohort. In conclusion, we were able to identify a novel necroptosis-related prognostic gene signature for CESC, which could provide new clues for prediction prognosis in patients with CESC.

We further investigated the association between the expression of the six NRGs and various molecular criteria as well as the different clinical parameters in CESC patients. Our findings showed that the expression levels of most of the six prognostic NRGs were associated with tumor stages, histological type, and therapy outcome in CESC patients. Moreover, the NRGs were shown to be involved in tumor development and progression.

Our verification experiment via IHC showed that expression levels of CYBB, IL1A, IL1B, and SLC25A5 proteins in CESC tissues were significantly higher than in adjacent non-tumor tissues, while CAMK2A and TICAM2 protein expression levels were found to be greater in normal tissues than in cancerous tissues. These results matched our findings in TCGA CESC database and further confirmed the reliability of our models. On the other hand, we investigated the relationship between risk score of our necroptosis risk scoring signature and expression levels of TP53, PIK3CA, PTEN, STK11, and KRAS; this five driver genes were previously shown to drive CESC. This finding provided clues and targets for further investigation.

TILs were shown to be independent predictors of tumor stage, grade, and lymph node status in various cancer types [37]. In line with this, we explored the correlation between the expression levels of the six prognostic NRGs and immune cell infiltration in CESC. Our research revealed considerable relationships between the expression levels of these six prognostic NRGs and immune cell infiltration in CESC. Our findings showed that these six NRGs, especially CYBB, IL1A, and IL1B might play a vital function in modulating the infiltration of immune cells in CESC. Our study also explored the correlation between the six NRGs, TMB, and MSI in CESC. We assumed that IL1B and TICAM2 could serve as biomarkers for evaluating the efficacy of immunotherapy in CESC. Moreover, we found that the expression levels of CAMK2A, CYBB, IL1A, IL1B, and TICAM2 were significantly correlated with various immunological checkpoints in CESC. These findings suggest that our study may contribute to the development of immunotherapeutic strategies in CESC. Taken together, our findings pointed to a possible implication of the six NRGs signature in tumor immune evasion and antitumor immunity which mediates the carcinogenic processes in CESC.

Regarding the relationship between immune cell infiltration and the six prognostic NRGs, it was interesting to note that different approaches produced inconsistent results. The following reasons may explain this inconsistency. In spite of the fact that flow cytometry, immunohistochemistry, and single-cell sequencing can be used to assess the immune status of a tumor sample, there are limitations to each of them that prevent them from being widely used. As a result, we used computational methods to analyze bulk RNA-sequencing data in order to assess immune cell composition. Firstly, there were some variations between the computer-based algorithms and the actual situation. Secondly, tumor immune cell infiltration mechanisms are complex, and small sample sizes and intratumor heterogeneity inevitably influence them. At the end, these methods use different algorithms, each of which has its own advantages and disadvantages.

In summary, we constructed a novel necroptosis-related gene signature, which includes CAMK2A, CYBB, IL1A, IL1B, SLC25A5, and TICAM2, for predicting the prognosis of CESC patients. The expression of these six NRGs in CESC were validated by immunohistochemistry experiments. We also demonstrated that the expression of these six NRGs was significantly related to TILs, chemokines, TMB, MSI, and immunological checkpoints in CESC. Our findings suggested that the six NRGs may be key players in CESC carcinogenesis through their actions in tumor immune cell infiltration. Nonetheless, further fundamental studies and extensive clinical trials are necessary to validate our findings.

## Supplementary Information

**Additional file 1: Table S1.** Clinical characteristics of patients with CC. Abbreviations: CC, Cervical carcinoma.

**Additional file 2: Table S2.** 159 NRGs from KEGG in CESC. Abbreviations: CESC, Cervical squamous cell carcinoma and endocervical adenocarcinoma.

**Additional file 3: Table S3.** 25 NRGs among the DEGs between CESC and normal samples. Abbreviations: CESC, Cervical squamous cell carcinoma and endocervical adenocarcinoma.

**Additional file 4: Table S4.** Six NRGs associated with prognosis in CESC. Abbreviations: CESC, Cervical squamous cell carcinoma and endocervical adenocarcinoma.

## Data Availability

All the datasets were retrieved from the publishing literature, so it was confirmed that all written informed consent was obtained.

## Abbreviations

CC: Cervical carcinoma
CESC: Cervical squamous cell carcinoma and endocervical adenocarcinoma
OS: Overall survival
TCGA: The Cancer Genome Atlas
NRGs: Necroptosis-related genes
KEGG: Kyoto Encyclopedia of Genes and Genomes
GO: Gene ontology
LASSO: Least absolute shrinkage and selection operator
ROC: Receiver operating characteristic
TMB: Tumor mutation burden
MSI: Microsatellite instability
DAMPs: Damage-associated molecular patterns
RIPK1/RIPK3: Receptor-interacting serine/threonine protein kinase 1/3
MLKL/pMLKL: Mixed lineage kinase domain-like protein
KM plotter: Kaplan-Meier plotter
HR: Hazard ratio
CI: Confidence interval
PR: Progesterone receptor
TIMER: Tumor Immune Estimation Resource
TISIDB: Tumor-Immune System Interaction Database
TILs: Tumor-infiltrating lymphocytes
FC: Fold-change
BP: Biological process
MF: Molecular function
PFS: Progression-free survival
DSS: Disease-specific survival
AUCs: Area under the curves
Tregs: T cell regulatory
NK cell: Natural killer cell
DCs: Dendritic cells
