# Supplementary material for: Identification of a necroptosis-related prognostic gene signature associated with tumor immune microenvironment in cervical carcinoma and experimental verification
Source: World J Surg Oncol. 2022 Oct 17;20:342. doi: 10.1186/s12957-022-02802-z (PMC9575203; doi:10.1186/s12957-022-02802-z)
Supplement: Supplementary file 1 — Additional file 1: Table S1. Clinical characteristics of patients with CC. Abbreviations: CC, Cervical carcinoma. [file 12957_2022_2802_MOESM1_ESM.docx]

**Table S1** Clinical characteristics of patients with CC

| Characteristic | No.of patients(%) |
| --- | --- |
| n | 22 |
| **Age, n (%)** |  |
| ≤45 | 7(31.8%) |
| >45 | 15 (68.2%) |
| **Histological type, n (%)** |  |
| Squamous cell carcinoma | 16 (72.7%) |
| Adenocarcinoma | 6 (27.3%) |
| **Clinical stage, n (%)** |  |
| Ⅰ-Ⅱ | 19 (86.4%) |
| Ⅲ-Ⅳ | 3 (13.6%) |
| **Lymph node metastasis, n (%)** |  |
| No | 19 (86.4%) |
| Yes | 3 (13.6%) |
| **Invasion depth, n (%)** |  |
| low-muscle | 14(63.6%) |
| depth muscle | 8 36.4%) |
| **Infiltrating glands, n (%)** |  |
| No | 11 (50%) |
| Yes | 11 (50%) |

Abbreviations: CC, Cervical carcinoma.
